# Rapid, chemical-free breaking of microfluidic emulsions with a hand-held antistatic gun

**DOI:** 10.1063/1.4995479

**Published:** 2017-07-20

**Authors:** Mohsen Karbaschi, Payam Shahi, Adam R. Abate

**Affiliations:** 1 Department of Bioengineering and Therapeutic Sciences, California Institute for Quantitative Biosciences, University of California, San Francisco, California 94158, USA; 2 Chan Zuckerberg Biohub, 499 Illinois St., San Francisco, California 94158, USA

## Abstract

Droplet microfluidics can form and process millions of picoliter droplets with speed and ease, allowing the execution of huge numbers of biological reactions for high-throughput studies. However, at the conclusion of most experiments, the emulsions must be broken to recover and analyze their contents. This is usually achieved with demulsifiers, like perfluorooctanol and chloroform, which can interfere with downstream reactions and harm cells. Here, we describe a simple approach to rapidly and efficiently break microfluidic emulsions, which requires no chemicals. Our method allows one-pot multi-step reactions, making it useful for large scale automated processing of reactions requiring demulsification. Using a hand-held antistatic gun, we pulse emulsions with the electric field, coalescing ∼100 *μ*l of droplets in ∼10 s. We show that while emulsions broken with chemical demulsifiers exhibit potent PCR inhibition, the antistatic-broken emulsions amplify efficiently. The ability to break emulsions quickly without chemicals should make our approach valuable for most demulsification needs in microfluidics.

## INTRODUCTION

Droplet microfluidics is a rapidly growing subfield of microfluidics in which picoliter-volume droplets are used for applications in biology, chemistry, and physics.^1–3^ Though simple, the technology is amazingly general, with applications in systems biology (genetic interaction studies and single cell transcriptomics),^4,5^ synthetic biology (enzyme and microbe evolution),^6^ structural biology (protein crystallization and sequence-function mapping),^7,8^ microbiology (rare cell cultivation and sequencing),^9^ tissue engineering (hydrogel encapsulation and cell delivery),^10^ and as general analytical tools (digital droplet PCR, digital ELISA, and nucleic acid cytometry).^11–14^ All of these applications leverage the ability of microfluidic devices to form, merge, inject, analyze, and sort huge numbers of droplets quickly and efficiently.^14–19^ In addition, recent advances have boosted throughput further, increasing droplet generation to megahertz^14^ and droplet sorting to over 30 kHz,^15^ providing unprecedented potential for characterizing systems comprehensively.

Often, the final step in an experiment is to recover the material from the millions of droplets produced by a microfluidic workflow. This is needed, for example, to recover barcoded nucleic acids for single cell transcriptome sequencing or plasmids encoding gene libraries for sequence-function mapping.^4,8^ One approach is to electrically coalesce droplets with a stream of flowing aqueous phase in a microfluidic device.^20^ This, however, requires the construction and operation of a microfluidic device, which is overly complicated for such a simple task. An easier method is to chemically break emulsions with demulsifiers, such as chloroform or perfluorooctanol (PFO); these chemicals displace surfactants from the oil-water interfaces of the droplets, making them unstable.^21,22^ Some demulsifiers, however, can be partially soluble in aqueous phases, where they can interact with hydrophobic residues of important compounds, like proteins. Consequently, they can inhibit cell growth and biological reactions, especially involving enzymes.^23^ A superior method for recovering the contents of aqueous droplets would quickly break an emulsion without the need of chemicals.

In this paper, we describe quick and efficient breaking of microfluidic emulsions without the use of chemicals. Using a hand-held antistatic gun, we pulse emulsions with the electric field, causing them to merge into a large, coalesced phase (supplementary material, movie). The emulsion progressively coalesces with each pulse of the field, with complete breaking of ∼100 *μ*l of emulsion in ∼10 s. Because no interfering chemicals are added, the recovered material is pristine and can be subjected to additional biochemical analysis, including involving enzymes. We show that while PCR amplification of the material recovered by perfluorooctanol demulsification is strongly inhibited, electric demulsification yields efficient, uninhibited amplification. The method is cost-effective, requiring only a $100 static gun with a working lifetime of years. It is general allowing breaking of emulsions comprising different polar and nonpolar phases, including fluorinated, silicone, and hydrocarbon oils. Its speed and simplicity, combined with the unadulterated nature of the recovered material, should make it the method of choice for breaking microfluidic emulsions.

## MATERIALS AND METHODS

**FIG. 1.**
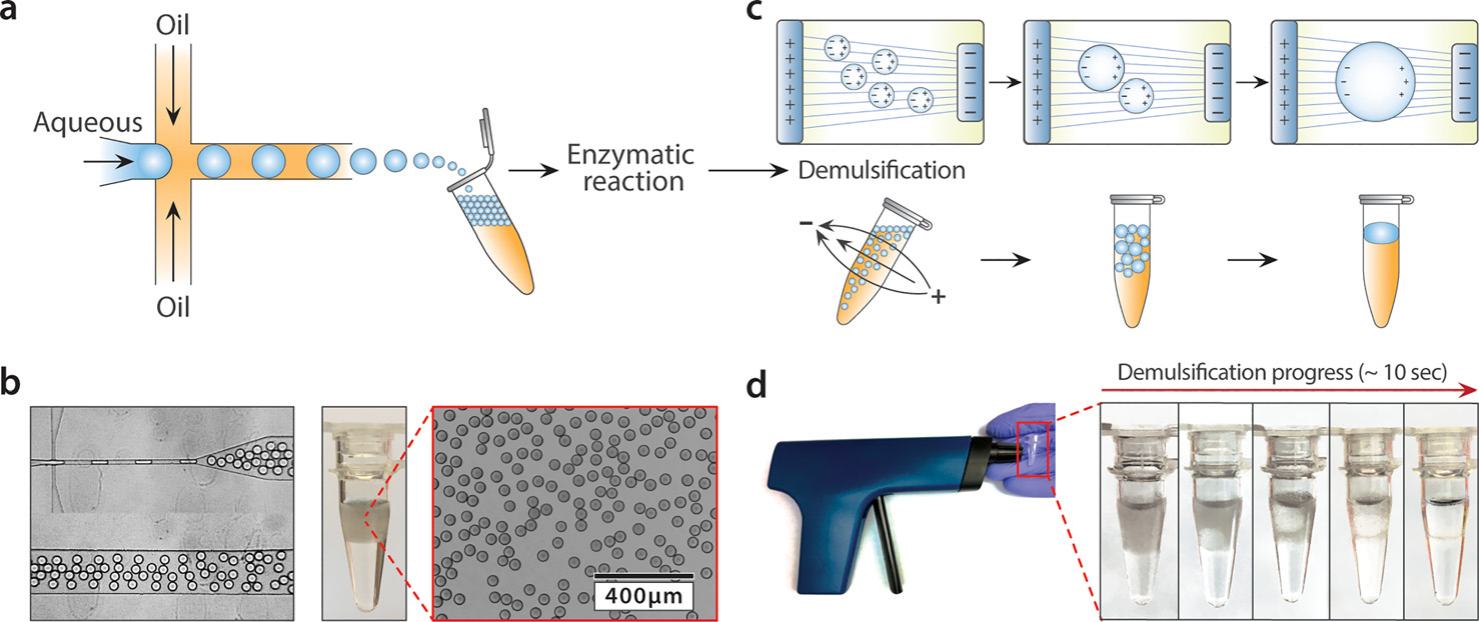
Demonstration of microfluidic emulsification and electric demulsification. (a) Cartoon of microfluidic flow-focusing water-in-oil droplet generation and collection. (b) Image of the PDMS flow-focus device generating water-in-oil droplets, collected into a tube, and optical microscope image. (c) Illustration of electric-coalescence of droplets due to polarization and electrically induced thin film instability. (d) Image of the antistatic gun (Milty Pro Zerostat 3, Armourhome) and an emulsion in a PCR tube before and after electric demulsification.

The devices made of polydimethylsiloxane (PDMS) are fabricated by soft lithographic techniques.^24^ SU-8 3025 (MicroChem, Newton, MA, USA) is spin-coated on a 3 inch silicon wafer and patterned via ultraviolet exposure through a photo transparency mask. The wafer is developed by submerging in 1-methoxy-2-propyl acetate and used to mould the PDMS device. The inlet and outlet holes are punched by a 0.75 mm biopsy core. The device is bound to a glass substrate by treating with oxygen plasma. The device channels are made hydrophobic by treatment with Aquapel. For drop formation, a flow focusing configuration is used.^25^ qPCR mix drops of 45 *μ*m in diameter are generated in hydrofluoroether (HFE, 3M™ Novec™ 7500 Engineered Fluid) that contains a 2% PEG-PFPE amphiphilic block copolymer surfactant (Ran Biotechnologies). The emulsions are made using a flow focus droplet generator with nozzle dimensions of 30 *μ*m at flow rates of 300 *μ*l/h and 900 *μ*l/h for the aqueous and oil, respectively. qPCR is performed using Maxima SYBR Green/ROX qPCR Master Mix (Thermo Fisher Scientific). qPCR primers are designed to amplify multiple regions of yeast chromosome 11 DNA from 125 pg/*μ*l of purified yeast genomic DNA (Millipore). Primer sequences are as follows: Set1: forward 5′ TGT TAC CCA ATG ACG ATG ACT AC 3′ and reverse 5′ CTC CAA CGA GCA CCG AAT TA 3′. Set2: forward 5′ GCA GGG CTT TCC TCG ATA TAA 3′ and reverse 5′ GAG TGA TCG CCG TAC AGA TAA G 3′. Set3: forward 5′ CTG AGC CCT CAG TAA CCA TTC 3′ and reverse 5′ GCC TAT CCG ACT GCA CTT TAT 3′. PCR parameters: 95 °C for 10 min, 40 cycles of 95 °C for 15 s, 55 °C for 30 s, 72 °C for 17 s. qPCR was performed using an Mx3005P qPCR System (Agilent Technologies). Electrical coalescence is achieved using an antistatic gun [Milty Pro Zerostat 3, Armourhome, shown in Fig. 1(d)].

## RESULTS AND DISCUSSION

Droplet microfluidics performs biological assays by partitioning samples into picoliter aqueous droplets suspended in carrier oil. Droplet partitioning is often accomplished with a cross-junction generator, in which the aqueous phase is segmented into droplets with oil [Fig. 1(a)]. Due to the small channels, flows are laminar and interfacial tension dominates over viscous forces, allowing controlled generation of monodisperse droplets at kilohertz rates [Fig. 1(b)]. Microfluidic techniques have advanced beyond forming droplets; they can also merge, picoinject, and sort them, in an order customized to the specific task. Droplets can also be incubated and thermally cycled, as needed for cell culture, enzymatic reactions, and DNA amplification via PCR. The net result of such “workflows” is to produce copious numbers of droplets, often in the tens of millions. At the conclusion of a workflow, the final step is usually to recover the contents of the droplets. For example, when performing digital droplet multiple displacement amplification, this is required to recover the amplified DNA for sequencing.^26^ This requires “breaking” the emulsion, which is normally accomplished with a chemical demulsifier. For fluorinated oil emulsions, which currently dominate the field due to their gas solubility, stability, and compatibility with PDMS devices, perfluorooctanol (PFO) is usually used.^21,22^ PFO, however, like most demulsifiers, displaces surfactants from the water-oil interface; this allows potential interactions of the interface with compounds dissolved in the aqueous phase, like amino and nucleic acids. While these effects can be mitigated by adding even more chemicals, a simpler and superior method would be to break the emulsion without the use of demulsifiers.

Electric fields are incredibly effective for coalescing conductive droplets suspended in insulating oil. Indeed, unintended coalescence of aqueous emulsions by static electricity on tubes, gloves, and devices is a common failure mode in microfluidic labs. In the presence of the electric field, conductive droplets polarize, creating attractive forces that induce coalescence via a thin film instability [Fig. 1(c)].^27^

Electrocoalescence is, thus, a general way to merge droplets in different oils. To use this as a controlled coalescence technique, we require a means of generating electric field where and when it is needed, as easily as possible. A simple device for generating focused electric field is an anti-static gun [Fig. 1(d)]. The gun consists of a housing with a ∼1 cm tip from which charged ions are ejected with each pull of the trigger. It is normally used to neutralize static charge buildup on insulating surfaces, like electronics, optical components, and vinyl records. The principle of emulsion coalescence with the antistatic gun is the same as for electrocoalescence^28^ and picoinjection^29^ in microfluidic channels. Therefore, similar to these techniques, our method applies to different emulsion types regardless of surfactant composition or concentration. Here, we use it to break an emulsion, pulsing droplets in a PCR tube with electric field by repeatedly pulling the trigger. Several pulls are required to completely break the emulsion, usually taking ∼10 s total for ∼100 *μ*l of 45 *μ*m in diameter droplets [Fig. 1(d), supplementary material (movie)]. It is helpful to position a conductor (like a gloved finger) on the backside of the tube to act as a sink for electric field and, thus, concentrate the field through the tube.

Aqueous droplets, particularly when containing salty biological buffers, are conductive and normally either cream (in denser fluorinated oil) or sediment (in less dense hydrocarbon and silicone oil), forming a close pack. However, close-packed emulsions are hard to break because surface droplets shield buried droplets from the field. To allow the field to penetrate deep into the emulsion, the droplets must be unpacked, which is easily accomplished by gently rotating the tube in the presence of a gravitational field [Fig. 1(c)].

**FIG. 2.**
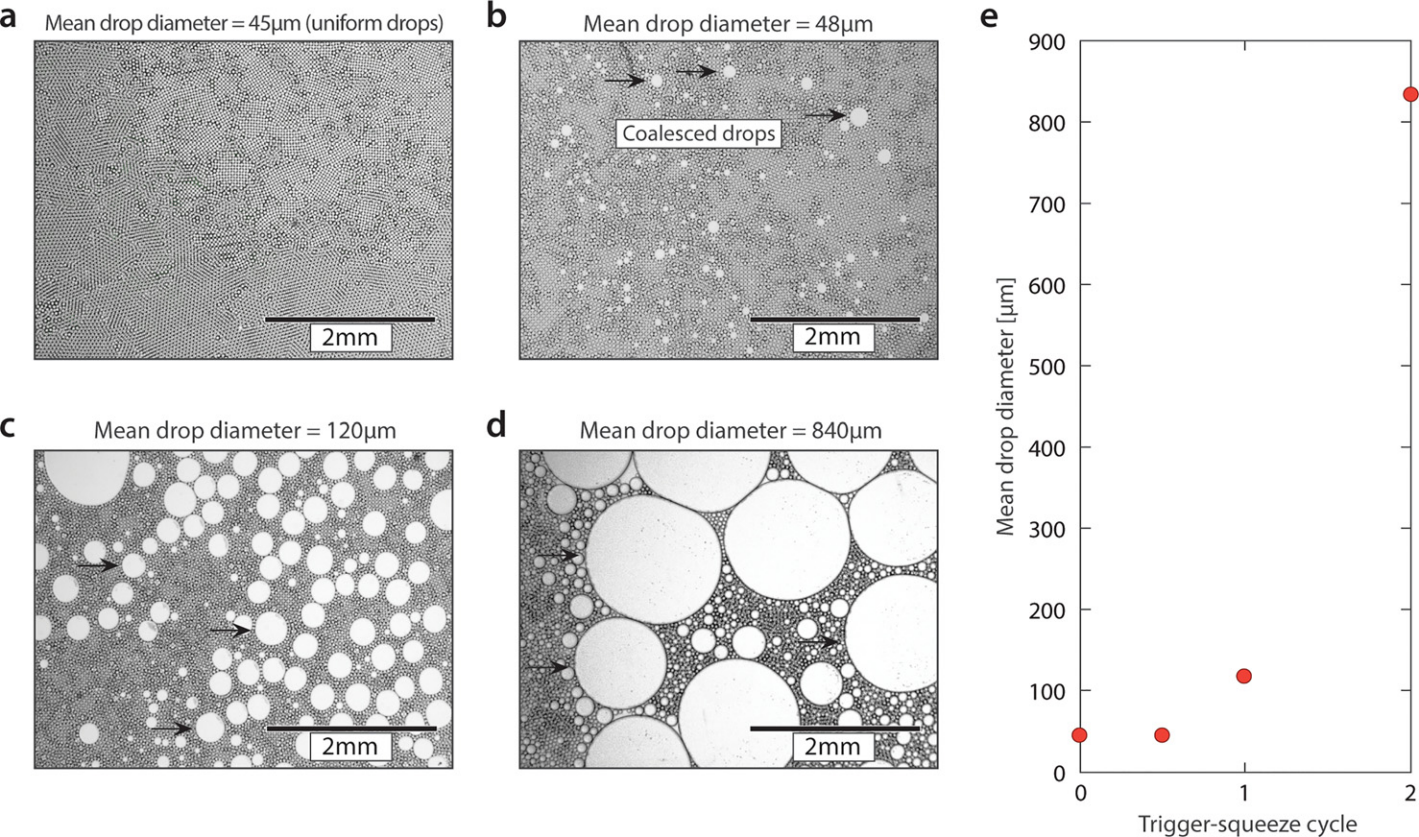
Demulsification progresses steadily with increasing electric field dose. (a) Microscopy image of freshly made water in oil emulsion and (b)–(d) with increasing electric field dose by squeezing the antistatic gun trigger. (e) Average droplet size increases rapidly with the trigger pull, completely breaking 20 *μ*l of emulsion in ∼5 pulls.

To observe the dynamics of coalescence in greater detail, we image samples of emulsion exposed to different numbers of pulses. Immediately after generation, the emulsion is monodisperse, since it was formed with microfluidics [Fig. 2(a)]. After a half-pulse cycle, there are already noticeable merged droplets [Fig. 2(b)], while additional cycles steadily increase the fraction of coalesced droplets and their size [Figs. 2(c) and 2(d)]. The average droplet volume grows quickly with the number of pulses [Fig. 2(e)], which is reasonable since the volume of a coalesced droplet is equal to the sum of the droplets that merged to form it. This allows millions of droplets to be completely merged in just a few cycles of the field.

The number of pulses required to completely coalesce an emulsion may vary depending on the specific chemical formulation. For example, the starting droplet size and conductivity, total volume of emulsion to coalesce, and permittivity and viscosity of the oil are all important factors. In general, one can expect complete coalescence of 100 *μ*l of emulsion with under 15 pulse cycles, which takes under 15 s. Nevertheless, since the electric field dose is controlled by pulling the gun's trigger, it is straightforward to adjust dose to achieve complete breaking of an emulsion. In addition, electric fields are relegated to the surfaces of the conductive droplets, since charge rearranges on their surface to screen field from their bulk. Reagents within the drops are not exposed to the field as they are dissolved in conductive biological buffers and shielded by surface charge rearrangement; this protects important components in the droplets, like cells and proteins, making the approach gentle. This is like other approaches that use electric fields to merge and sort droplets that are benign with respect to dissolved biological components.^21,30,31^

**FIG. 3.**
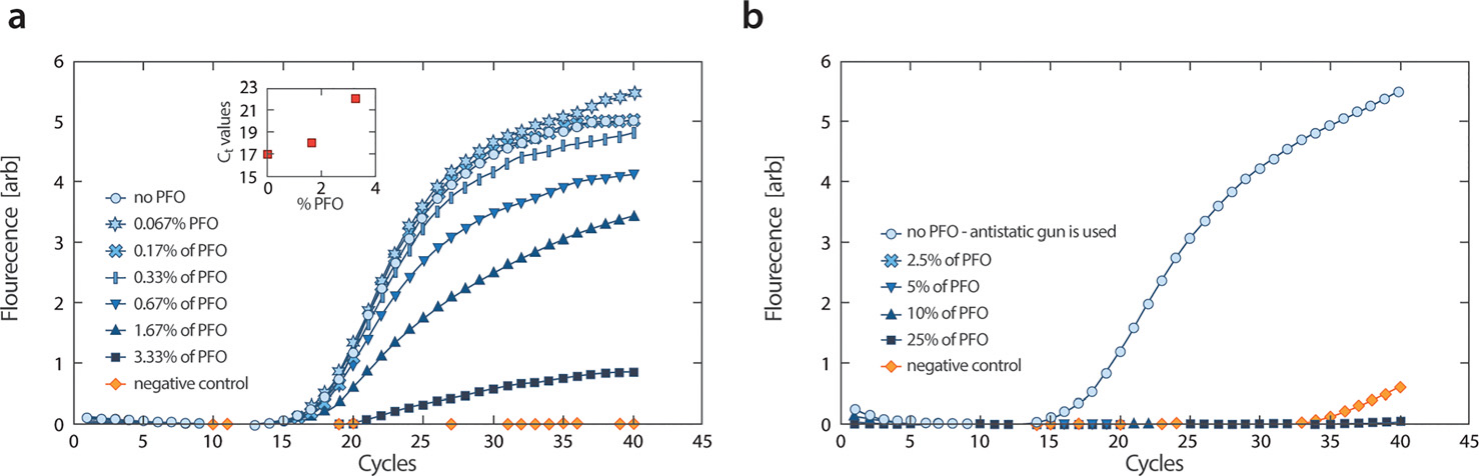
PCR is inhibited by the chemical demulsifier PFO but not electric demulsification. (a) qPCR data for droplets containing PCR reagents and DNA, suspended in oil with different concentrations of PFO; the droplets are thermally cycled with PFO present while their fluorescence is measured. While emulsions devoid of PFO amplify rapidly, as little as 3.3%, well below the 25% normally used for demulsification, completely inhibits the reaction. (b) Comparison of PCR amplification in the bulk for the material recovered by PFO and electric demulsification. While the material recovered by electric demulsification amplifies efficiently, PFO demulsification results in potent inhibition, with even the no-template negative control amplifying sooner.

To illustrate the gentle nature of the approach, we compare the efficiency of PCR amplification for emulsions subjected to PFO or electric demulsification (Fig. 3). We generate droplets containing 125 pg/*μ*l DNA targets and qPCR reagents. We add increasing concentrations of PFO, from 0 to 3.3%, and thermally cycle the emulsions while measuring fluorescence on a quantitative PCR machine. Prior to thermal cycling, we remove excess oil, so that the PCR tubes are filled with 20 *μ*l of emulsion droplets in 60 *μ*l of oil. While PCR is efficient and occurs rapidly when no PFO is present, the reaction becomes increasingly inhibited as PFO is added, with near complete inhibition at only 3.33% [Fig. 3(a)]—well below the ∼25% normally used to break emulsions; indeed, all of these emulsions are stable through thermal cycling with PFO present. This shows that minute amounts of PFO can significantly impact the efficiency of enzyme-based reactions [Fig. 3(a), inset].

In most uses of emulsion breaking, however, the demulsifier is added at the very end, when the emulsion needs to be coalesced and the droplet contents recovered; it is not added during incubation of the in-droplet reaction. To investigate whether PFO can impact reactions occurring after demulsification, we perform other experiments in which we encapsulate DNA and qPCR reagents and attempt to break the emulsions by adding PFO at 25% concentration, followed by vortexing. We then dilute the remaining oil values ranging between 25% and 2.5%. We also break one of the emulsions with the antistatic gun as a comparison and thermally cycle all tubes while measuring fluorescence on a qPCR machine. We find that while the electrically coalesced emulsion amplifies early and efficiently, all the PFO-coalesced emulsions are completely inhibited, with even the no-DNA control electrically coalesced emulsion amplifying earlier [Fig. 3(b)]. These experiments illustrate the potential for unintended interaction of demulsifiers on enzyme-based reactions occurring after emulsion breaking. While this is just one reaction, other enzymes may also be affected by demulsifiers. One approach to avoid such undesired interactions is to transfer the aqueous phase to a clean tube before performing the next reaction. This, however, necessitates additional handling steps that increase the risk of contaminating the sample, for example, due to environmental DNA or microbes. Ideally, one would add no unnecessary chemicals to the material that must be subjected to follow-on reactions, which is possible using electric demulsification.

## CONCLUSIONS

We have demonstrated a simple method to break microfluidic emulsions using a hand-held antistatic gun. Our method requires no chemicals and is fast and simple. The method applies to other water-in-oil emulsion formulations, regardless of surfactant composition, concentration, or oil type. The gun costs $100 and can coalesce emulsions over an operating lifetime of years, making it extremely cost effective. In addition, chemical demulsifiers can have unintended interactions with the recovered material, as we have shown, inhibiting follow-on PCR far below the concentrations required for effective demulsification. The ability to break emulsions without adding chemicals maintains the material in an unadulterated state for downstream analysis; this allows one-pot multi-step reactions without inhibition due to added demulsifiers. The simplicity, speed, and low cost of our approach should make it valuable for most droplet microfluidic demulsification needs.

## SUPPLEMENTARY MATERIAL

See supplementary material for a video of demulsification of a microfluidic emulsion with the antistatic gun.
